# Predictors of Women's Satisfaction with Hospital-Based Intrapartum Care in Asmara Public Hospitals, Eritrea

**DOI:** 10.1155/2017/3717408

**Published:** 2017-12-28

**Authors:** Meron Mehari Kifle, Filmon Abraham Ghirmai, Soliana Amanuel Berhe, Winta Sium Tesfay, Yodit Teklemariam Weldegebriel, Zebib Tesfamariam Gebrehiwet

**Affiliations:** ^1^Department of Epidemiology and Biostatistics, School of Public Health, Asmara College of Health Sciences, Asmara, Eritrea; ^2^School of Nursing, Asmara College of Health Sciences, Asmara, Eritrea; ^3^Ministry of Health, Asmara, Eritrea

## Abstract

**Background:**

Exploring patient satisfaction contributes to provide quality maternity care, but there is paucity of epidemiologic data in Eritrea.

**Objectives:**

To determine the predictors of women's satisfaction with intrapartum care in Asmara public maternity hospitals in Eritrea.

**Methods:**

A cross-sectional study among 771 mothers who gave birth in three public Hospitals. Chi-square tests were done to analyze the difference in proportion and logistic regression to assess the predictors of satisfaction with intrapartum care.

**Results:**

Overall, only 20.8% of the participants were satisfied with intrapartum service. The key predictors of satisfaction with intrapartum care were provision of clean bed and beddings (AOR = 18.87, 2.33–15.75), privacy during examinations (AOR = 10.22, 4.86–21.48), using understandable language (AOR = 8.72, 3.57–21.27), showing how to summon for help (AOR = 8.16, 4.30–15.48), showing baby immediately after birth (AOR = 8.14, 2.87–23.07), control of the delivery room (AOR = 6.86, 2.65–17.75), receiving back massage (AOR = 6.43, 3.23–12.81), toilet access and cleanliness (AOR = 6.09, 3.25–11.42), availability of chairs for relatives (AOR = 5.96, 3.14–11.30), allowing parents to stay during labour (AOR = 3.52, 1.299–9.56), and request for permission before any procedure (AOR = 2.39, 1.28–4.46).

**Conclusion:**

To increase satisfaction with intrapartum care, maternity service providers need to address the general maternity ward cleanliness, improve the quality of physical facilities, and sensitize health providers for better communication with clients. Policy makers need to adopt strategies that ensure more women involvement in decision making and consideration of privacy and reassurance needs during the whole delivery process.

## 1. Background

With the increasing need of client-centered care, there has been a growing consensus that patient service quality perceptions are critical for maintaining and monitoring the quality of health care [1]. Women's satisfaction with maternity service is often associated with the quality of intrapartum care, as the nature of the support given during labour and childbirth is reflective of a positive birth experience [2].

Patient satisfaction measures the ability of services to meet consumers' expectations [3] and is an important determinant of the choice of health facility and its future utilization [4–6]. Satisfaction is a complex and multidimensional concept comprising structure, process, and outcome of care [7]. Assessing maternal satisfaction helps in the provision of a more responsive and culturally acceptable care which can lead to an increase in service utilization and better outcomes. Patient satisfaction also ensures that the views of the users are taken into account and helps to develop culturally appropriate services [8]. Furthermore, satisfied clients are more likely to return in the future [8], adhere to health provider's recommendations [9], and recommend the institution to their friends and relatives, effecting an increased demand for the service [10].

In Eritrea, three-fourths of all health facilities provide maternal and child health services including antenatal care, delivery services, postnatal care, immunization services, growth monitoring, health education, and family planning. Consequently, significant achievements have been recorded in maternal and child health indices over the past two decades. Infant mortality rates per 1,000 live births have decreased from 92 in 1990 to 58 in 2000 and to 37 in 2012. Eritrea has already exceeded its MDG-5 target of maternal mortality ratio of 425 per 100,000 live births [11]. The maternal mortality ratio declined from 1,700 per 100,000 live births in 1990 to 670 in 2000 and 380 in 2013. Furthermore, access to emergency obstetric care services increased by more than 300 percent between 1995 (21%) and 2013 (88%) [12].

Despite these improvements, however, significant deficits in the provision of quality maternity services continue to remain a considerable challenge. The current proportion of births with skilled attendance in Eritrea is 34.1%, a figure which has not increased much since 1995 (21%) [13]. The majority of maternity care services are provided by nurses, midwives, and health assistants. Inadequate staffing and physical infrastructure, increasing maternal health care utilization, low use of family planning, and overloaded health care providers with limited training have also resulted in compromised quality of maternal health services [14, 15].

In this context, studies that explore women's satisfaction with intrapartum care are timely and relevant. Therefore, the objective of this study was to determine the factors associated with women's satisfaction with labour and childbirth services in public hospitals in Eritrea. Specifically, this study sought to examine satisfaction in terms of sociodemographic characteristics and with four dimensions of care: provision of physical facilities, provision of consumables, pain management methods, and communication patterns of healthcare providers.

## 2. Methods

### 2.1. Study Design

A descriptive cross-sectional design was used for this study.

### 2.2. Study Area

This study was conducted in Orotta Maternity National Referral Hospital (OMNRH), Edaga Hamus Hospital (EHH), and Villagio Community Hospital (VCH). These hospitals were selected because they generally have the patient's profile that is characteristic of most public hospitals in Eritrea. OMNRH is the busiest maternity center with high turnover of mothers giving birth. This hospital has about 8000 normal deliveries annually, representing 34% of the total national normal deliveries. OMNRH is a teaching hospital and accommodates medical students, nurses, nurse midwives, and others. Edaga Hamus Hospital, which is located in North East of Asmara, was renovated in April 2014 and had a total of 467 deliveries in that year. In 2015, delivery services were provided for about 1060 mothers. Villagio Community Hospital is the third public hospital that gives delivery service. It is located in North West of Asmara and started providing delivery service in June 2014. Annual HMIS report indicates that there were about 206 deliveries in 2015.

### 2.3. Study Participants and Sampling Technique

Using a temporal (period) sampling technique [16], 771 women (99.6% response rate) who gave birth at OMNRH, EHH, and VCH hospitals from March to May 2016 participated in the study. All women who delivered by spontaneous vaginal delivery successfully with or without episiotomy and women who were on their immediate postpartum care during the study period were enrolled in the study. Women who were seriously ill, not consented to participate, and with incomplete data and women who experienced birth complications requiring admission to a special care were excluded.

### 2.4. Measures

The questionnaire was developed after an extensive review of the literature. The tool was modified and finalized according to the suggestions and recommendations of local experts (one gynecology and obstetrics specialist and lecturer at the Asmara College of Health Science, School of Nursing, two midwifery practitioners at the National Maternal and child health referral hospital, and a senior statistician at the Ministry of Health) and the research team. Content validity was secured through in-depth interviews and critical appraisal of the data collection instrument.

The final questionnaire had two sections. The first part included questions about the respondent's age, religion, level of education, parity, mode of delivery, and marital status. The second section was a scale measuring women's satisfaction with the four dimensions of intrapartum care. The scale was generated by summing up the mean and standard deviation scores of the four subscales.

The subscale items were formulated from extensive literature review and expert input. The subscale scores were constructed from responses to individual questions. They were summarized using the average (mean) score plus one standard deviation (SD). Scores above the mean and one standard deviation were considered satisfied [17, 18]. Subscale one contained items related to the provision of physical facilities (6 items). The second subscale included questions regarding the provision of consumables (4 items). The third subscale included questions about women's satisfaction with pain management methods (3 items). The last subscale contained questions about the communication patterns of health care providers (7 items).

Participants were asked to rate their satisfaction with intrapartum care on a five-point Likert scale ranging from strongly disagree (1) to strongly agree (5). The satisfaction scale had a reliability score of 0.702. To address for face validity, the questionnaire was piloted with a group of 20 childbearing women in Villagio Community Hospital.

### 2.5. Data Collection Method

After brief explanation of the study objectives, the respondents were assured about the confidentiality and anonymity of their responses. Written consent was then obtained to participate in the study. Four final year nursing students approached the women and made interviews in the wards behind closed curtains for privacy. After completing the interviews, the filled questionnaires were checked for completeness, consistency, and the presence of outliers. A database was developed in CSPro 6.2 and pretested before the start of data entry. Data entry was supervised by the researchers, and any suspect data were cross-checked against hard copies of the questionnaires.

### 2.6. Data Analysis Method

Data were analyzed using the Statistical Package for the Social Sciences (SPSS) version 21. The properties of the instrument were assessed using Cronbach's alpha for reliability (0.702). Relationships between dependent variable (satisfaction with intrapartum care) and independent variables (demographic, obstetric, and intrapartum care indicator variables) were examined using chi-square tests. Statistically significant variables were then dichotomized. Responses of “very satisfied” and “satisfied” were classified as “satisfied” and responses of “very dissatisfied,” “dissatisfied,” and “neutral” as “unsatisfied.” Neutral responses were categorized as dissatisfied because the interview was done in the hospitals, and interviewer or social desirability bias might have had an effect in disclosing their dissatisfaction [17, 19]. Finally, to identify predictors of satisfaction with intrapartum care, binary and multiple logistic regression analyses were done. Statistical significance was set at *P* < 0.05.

## 3. Results

### 3.1. Socio-Demographic Profile

Totally, 771 women agreed to participate in the study. The mean age of the participants was 28.84 ± 5.877. Almost half (54.9%) of the respondents were between 25 and 34 years old. More than half (69.6%) were multiparous, 56.2% had spontaneous vaginal delivery, and 86.9% were married. Participant characteristics are shown in Table 1.

### 3.2. Satisfaction with Intrapartum Care

The total mean satisfaction score was 72.36 (SD ± 10.56; range 43–107). Scores of ≥82 were considered positive towards increased satisfaction with intrapartum care. Only 20.8% (*n* = 161) of the participants scored ≥ 82. The remaining 79.1% (*n* = 610) women scored lower, suggesting dissatisfaction with the four dimensions of intrapartum care.

The mean subscale score for the provision of physical facilities was 26.78 (SD ± 4.371), range 8–35. Scores of ≥31 were considered positive. Only 13.1% of the participants (*n* = 135) scored ≥ 31. The remaining 86.9% (*n* = 636) scored low, suggesting dissatisfaction with the provision of physical facilities.

The mean subscale score for provision of consumables was 5.65 (SD ± 2.588), range 5–20. Scores of ≥8 were considered positive. Only 16.6% of the participants (*n* = 128) scored ≥ 8. The remaining 83.4% (*n* = 643) scored lower, suggesting dissatisfaction with the provision of consumables.

The mean subscale score for pain management methods was 7.25 (SD ± 2.564). Scores of ≥10 were considered positive (range 3–15). Only 18.4% of the participants (*n* = 142) scored ≥ 10. The remaining 81.6% (*n* = 629) scored lower, suggesting dissatisfaction with pain management methods.

The mean subscale score for communication patterns of health care providers was 30.14 (SD ± 5.573), range 12–40. Scores of ≥36 were considered positive. Only 22.3% of the participants (*n* = 172) scored ≥ 36. The remaining 77.7% (*n* = 599) scored lower, suggesting dissatisfaction with communication patterns of health care providers (Table 2).

### 3.3. Predictors of Satisfaction with Intrapartum Care

There was no association between women's sociodemographic data (age, education, religion, parity, and mode of delivery) and satisfaction with intrapartum care. Variables associated with the outcome variable were entered in a binary logistic regression analysis. The final multivariate logistic regression model showed eleven influential predictors of low satisfaction with intrapartum care. The results are as shown in Table 3.

From the first indicator (provision of physical facilities), women who reported that they were not given clean bed and beddings were more likely (AOR = 18.87) to be dissatisfied with intrapartum care. Similarly, women who reported poor toilet cleanliness and ease of access were more likely (AOR = 6.09) to be dissatisfied with intrapartum care. On the question of women's perceived control of the delivery room, respondents who reported that they had no control were more likely to be dissatisfied with intrapartum care. Moreover, unavailability of comfortable chairs for relatives was a positive predictor (AOR = 5.96) of low satisfaction with intrapartum care (Table 3).

From the second indicator (provision of consumables), all of the predictor variables did not have statistically significant association with the outcome variable and were excluded from the regression model. However, some significant findings did emerge. Majority (98.3%) of the respondents reported unavailability of water for showering, 95.8% reported sanitary pads were not provided after delivery, and more than two-thirds reported that adequate food and hot drinks were not provided.

In the third indicator (pain management methods), women who reported that they were not taught how to breath in deeply during severe pain and to rest when pain wore off were more likely to be dissatisfied with the care they received. Moreover, women who perceived that they were not given adequate privacy during examinations were ten times more likely (AOR = 10.22) to be dissatisfied with intrapartum care.

Women who did not receive adequate back massage were six times more likely (AOR = 6.43) to be dissatisfied than the ones who felt they did receive adequate back massage (Table 3).

From the fourth indicator (communication patterns of health care providers), women who reported that the staff did not ask for permission before any procedure were more likely (AOR = 2.39) to be dissatisfied with intrapartum care. Moreover, women who perceived that they were not given adequate privacy during examinations were ten times more likely (AOR = 10.22) to be dissatisfied with intrapartum care. Women reporting that the staff did not use a language that they could easily understand were at higher odds (AOR = 8.72) to be dissatisfied to those who said they did. Women who reported that the staff did not greet them with a smile and give a warm welcome, staff not showing a genuine interest in their well-being, staff not showing how to summon for help from them, not allowing relatives to stay during labour, and not showing baby immediately after birth were also important positive predictors of low satisfaction with intrapartum scale (Table 3). As an overall evaluation of future maternity care utilization, participants were asked if they would return to the hospital next time, and 83.4% responded favorably. Moreover, 86.1% of the women would recommend it for friends or relatives.

## 4. Discussion

Overall, only 20.8% (*n* = 161) of the participants were satisfied with intrapartum care. This rate was very low compared to study reports from Sri Lanka (48%) [20], Kenya (56%) [21], Côte d'Ivoire (92.5%) [22], or Ethiopia (81.7%) [23]. This variations may be due to a real difference in the quality of services provided, expectation of mothers, type of health facilities, or a combination of them [1, 9].

Worldwide, available findings regarding the association between demographic variables and satisfaction with intrapartum care are mixed, with some studies reporting that age, parity, and marital status were associated with satisfaction with intrapartum care [20, 24, 25]. Consistent with previous studies conducted in Kenya [21] and Jordan [17], this study found no association between women's sociodemographic data and satisfaction with intrapartum care.

Studies indicate that satisfaction with the physical environment is a significant predictor of women's overall satisfaction and positive experience of labour and delivery services [26, 27]. Similar to the findings of Lumadi and Buch [28], this study found higher rate of satisfaction with the cleanliness of the delivery environment (cleanliness of bed and beddings). This could be due to the fact that the cleaners are strictly supervised by the nurses in charge of the wards. On the other hand, more than half of the respondents were dissatisfied with toilet cleanliness and accessibility. This was similar with study findings from South Africa [29] and Kenya [30] but higher with one Ethiopian study [23]. In our setting, the reason for this could be due to the lack of adequate and continuous water supply, especially in Orotta and Edaga Hamus Hospitals. Toilet cleanliness is not well checked regularly by a responsible person in charge. This was exacerbated as the cleaners do not work on Sundays. Moreover, the high labour and delivery turnover are in lower proportions with the number of cleaners and hence cannot cope with the frequency of cleaning the available toilets.

Although variables related to the provision of consumables were not statistically associated with overall satisfaction, there was high dissatisfaction (83.4%) in all study cites. Regarding the provision of food and hot drinks, there was no regular provision during the three meal times. Women who deliver on weekends and women who came from rural areas highly commented on the provision of food and hot drinks. Similarly, sanitary pads were not adequately provided. Instead, women bring their own sanitary items at their own expense. Water scarcity is also a big challenge, especially in Orotta and Edaga Hamus Hospitals.

There has been growing evidence that events such as operative births, long and painful labour, inadequate pain relief, and increased obstetric interventions can adversely affect satisfaction with intrapartum care [24, 25, 31, 32, 34]. Our findings showed that only 18.4% of the participants were satisfied with pain management methods. This result coincides with previous studies [17, 28], where painful labour and being unhappy with the pain management methods were highly associated with low satisfaction rates.

Studies have reported that support from the health care providers during labour tends to improve childbirth outcomes and women's satisfaction [30]. Perceptions of satisfaction can also be affected through involvement in decisions about labour procedures [16, 18, 25]. This study found that women who were not given adequate privacy during examination were more likely to be dissatisfied with the services. Respondents who were not taught how to breathe in deeply during severe pain and to rest when pain wore off were more likely to be dissatisfied as were women who did not receive any back massage application. Although there is inadequate staff in the hospitals, breathing skills were not communicated even with the present number, suggesting it could well be the same even when staff number increases.

Interpersonal processes including perceived empathy, perceived technical competency, nonverbal communication, and patient enablement are believed to significantly influence patient satisfaction [25, 26, 33]. Most of the respondents were satisfied with the general approach of the staff during arrival which is consistent with one Eritrean study [35]. Almost half of the participants were not happy with the level of privacy of the wards. This finding was consistent with a study done in Kenya [30] but inconsistent with the other study [23]. Higher dissatisfaction in Orotta Hospital could be attributed to being a teaching hospital where a large number of students make it difficult to maintain privacy.

Satisfaction has been associated with interpersonal factors such as providing opportunities to have an active say during labour and birth, deciding when certain actions will be done, and being given information as to why such decisions are necessary [19, 25, 27, 31, 34]. In this study, high rate of dissatisfaction was disclosed on whether permission was requested before any procedure and the degree of involvement in decision making. This was alarming in view that, a recent systematic review has shown that participation in decision making and having an active say in decisions about one's care was an important dimension of satisfaction with health facility delivery [24]. One possible reason could be the health providers were not giving much attention to what women want or expect and give more priority in conducting the procedures before asking permission in advance. This issue is an emerging concern in Eritrea partly because for the past two decades, there has been a pressing need to make health services more accessible and the concept of client-centered service provision has been largely ignored until recently [11]. With increasing service utilization and awareness of the general public, the importance of optimal interpersonal communication and involvement of mothers in every decision making is likely to be a crucial dimension to maintain or increase the quality of health services.

Studies have shown that when mothers are assured that they can inquire anything about the whole delivery process and can summon health workers at any time, the overall satisfaction increases substantially [19, 25]. In the present study, almost half of the respondents were dissatisfied on staff showing how to summon them whenever they need. The weight of this issue was significant as women reporting that the health workers were not showing how to summon for help were eight times more likely to be dissatisfied. This was consistent with the findings of Nyaberi [30], where 56% of the respondents were dissatisfied.

Previous studies have suggested that many of the standard elements of quality of care have less effect on return behavior, whereas time and attention paid to health care users were the strongest predictors of returning to a health institution [19]. Our finding shows that more than two-thirds of all women would recommend the delivery care to their friends and family. Given that there was a relatively higher score on interpersonal communication of health care providers, this study suggests that although the proportion of mothers who were satisfied with delivery care was low, the respondents value the importance of health workers general behavior and interest in one's well-being. This attitude seems to override their ideal personal expectations and subsequent satisfaction with care. The findings also imply that there is plenty of room for substantial improvements for a more comprehensive, culturally acceptable, and quality intrapartum care.

## 5. Conclusion

The proportion of mothers who were satisfied with delivery care was low. Satisfaction and thus continued use can be achieved by addressing the general maternity ward cleanliness, improving the quality of physical facilities, and sensitizing health providers for better interpersonal communication with clients.

Health care professionals should also adopt strategies that ensure more women involvement in decision making, increasing individualized care, and support in labour and reassurance needs to improve satisfaction during the whole delivery process. Policy makers need to review the procedures and policies regarding childbirth practices in their hospitals. This information will help in planning and implementing appropriate strategies to assist women have a positive birth experience.

## Figures and Tables

**Table 1:** Sociodemographic characteristics of study participants (*n* = 771).

Variable	Total *N* (%)
*Age*	
15–24	211 (27.3)
25–34	425 (54.9)
35–44	131 (16.9)
45–49	4 (0.9)
*Parity*	
Primipara	234 (30.4)
Multipara	537 (69.6)
*Educational level*	
No education	24 (3.1)
Primary	95 (12.3)
Junior	249 (32.3)
High school	344 (44.6)
College level	59 (7.7)
*Mode of delivery*	
Spontaneous vaginal delivery	433 (56.2)
Assisted	9 (1.2)
Episiotomy	329 (42.7)
*Religion*	
Christian	632 (82)
Muslim	139 (18)
*Marital status*	
Single	96 (12.5)
Married	667 (86.5)
Divorced	4 (0.5)
Living together	3 (0.4)
Widowed	1 (0.1)
*Total*	**771 (100)**

**Table 2:** Satisfaction with intrapartum care scale, subscale items with satisfaction mean and standard deviation scores (*n* = 771).

Characteristic	Mean^∗^	SD^∗^
*Total scale* (*22 items*) Scores ≥ 82 considered satisfied	**72.36**	**10.56**
*Subscale 1: provision of physical facilities* (*6 items*), scores ≥ 31 considered satisfied	**26.78**	**4.371**
(1) Provided with clean and nicely decorated room	4.81	0.579
(2) Provided with clean bed and beddings	4.62	0.796
(3) Toilet ease of access and cleanliness	3.17	1.527
(4) Given a locker to keep personal items	3.74	0.860
(5) Chairs available for relatives	2.21	1.514
(6) Control of the delivery room	3.78	1.506
*Subscale 2: provision of consumables* (*4 items*), scores ≥ 8 considered satisfied	**5.65**	**2.588**
(1) Provision of hot water for showering	1.06	0.463
(2) Provision of sanitary pads after delivery	1.15	0.731
(3) Provision of adequate food	1.81	1.585
(4) Provision of hot drinks	1.61	1.420
*Subscale 3: pain management methods* (*3 items*), scores ≥ 10 considered satisfied	**7.25**	**2.564**
(1) Parents/sibling allowed to stay during labour	1.32	1.049
(2) Taught how to breath in deeply during severe pain and to rest when pain wore off	4.12	1.396
(3) Received adequate back massage	1.81	1.491
*Subscale 4: communication patterns of healthcare providers* (*7 items*), scores ≥ 36 considered satisfied	**30.14**	**5.573**
(1) Staff greeted with a smile	4.66	0.881
(2) Given adequate privacy during examination	3.32	1.569
(3) Staff requested for permission before any procedure	2.25	1.545
(4) Staff showed a genuine interest in one's well-being	4.42	1.002
(5) Staff used understandable language	3.79	1.550
(6) Staff showed how to summon for help	2.96	1.691
(7) Baby shown immediately after birth	4.19	1.522

^∗^The mean and standard deviation scores were computed with values as follows: strongly agree = 5; agree = 4; neutral = 3; disagree = 2; strongly disagree = 1.

**Table 3:** Bivariate and multivariate logistic regression analysis to identify predictors of low satisfaction with intrapartum care (*n* = 771).

	Odds ratio with 95% confidence interval	
	Crude OR	Adjusted OR
Provision of clean bed and beddings	30.47 (2.91–3.51)^∗^	18.87 (2.33–15.75)^∗^^∗^
Toilet ease of access and cleanliness	6.64 (3.38–13.03)^∗^^∗^	6.09 (3.25–11.42)^∗^^∗^
Given a locker to keep personal items	2.62 (0.70–9.74)	NA
Control of the delivery room	5.58 (2.00–15.54)^∗^^∗^	6.8 (2.65–17.75)^∗^^∗^
Parents/sibling allowed to stay during labour	7.78 (2.24–26.93)^∗^^∗^	3.52 (1.299–9.56)^∗^
Chairs available for your relatives	6.57 (3.30–13.08)^∗^^∗^	5.96 (3.14–11.30)^∗^^∗^
Taught breathing techniques	1.40 (0.48–4.11)	NA
Received adequate back massage	9.17 (4.22–19.91)^∗^^∗^	6.43 (3.23–12.81)^∗^^∗^
Given adequate privacy during examinations	11.52 (5.05–26.26)^∗^^∗^	10.22 (4.86–21.48)^∗^^∗^
Staff gave warm welcome	13.39 (2.24–19.91)^∗^^∗^	NA
Permission requested before any procedure	3.04 (1.53–6.06)^∗^^∗^	2.39 (1.28–4.46)^∗^^∗^
Staff showed a genuine interest	11.52 (5.05–26.26)^∗^^∗^	NA
Staff used understandable language	8.03 (3.11–20.72)^∗^^∗^	8.72 (3.57–21.27)^∗^^∗^
Staff showed women how to summon help	7.49 (3.79–14.78)^∗^^∗^	8.16 (4.30–15.48)^∗^^∗^
Baby shown immediately after birth	8.06 (2.60–24.99)^∗^^∗^	8.14 (2.87–23.07)^∗^^∗^

Bivariate model: *R*^2^ = 0.762; multivariate model: pseudo *R*^2^ = 0.726, ^∗^*P* < 0.05, ^∗^^∗^*P* < 0.001, NA = not applicable.
